# Cognitive function and return to work: protocol for a longitudinal study investigating cognitive endurance in patients with stress-related disorders in primary health care in Sweden

**DOI:** 10.1136/bmjopen-2026-125346

**Published:** 2026-11-06

**Authors:** Hanna Malmberg, Anastasiya Zakrevska, Magnus Johansson, Anna Nordberg, Elisabeth Björk Brämberg, Fabienne I M van Vliet, Birgit H.P.M. Donker-Cools, Frederieke G. Schaafsma, Anna Finnes

**Affiliations:** 1 Department of Psychology, Umeå University, Umeå, Sweden; 2 Department of Clinical Neuroscience, Division of Insurance Medicine, Karolinska Institutet, Stockholm, Sweden; 3 Academic Primary Health Care Center, Region Stockholm, Stockholm, Sweden; 4 Department of Clinical Neuroscience, Centre for Psychiatry Research, Karolinska Institutet, Stockholm, Sweden; 5 School of Public Health and Community Medicine, Institute of Medicine, University of Gothenburg, Gothenburg, Sweden; 6 Institute of Environmental Medicine, Unit of Intervention and Implementation Research for Worker Health, Karolinska Institutet, Stockholm, Sweden; 7 Department of Public and Occupational Health, Amsterdam UMC location University of Amsterdam, Amsterdam, The Netherlands; 8 Societal Participation and Health, Amsterdam Public Health Research Institute, Amsterdam, The Netherlands; 9 Department of Medical Psychology, Amsterdam UMC location University of Amsterdam, Amsterdam, The Netherlands

**Keywords:** Cognition, Stress, Psychological, Fatigue, MENTAL HEALTH, Primary Care

## Abstract

**Introduction:**

Patients with long-term stress-related disorders report cognitive symptoms across a broad range of domains. However, neuropsychological test results often do not align with these self-reported cognitive difficulties. One potential explanation is that patients may perform well during a single, undisturbed test session but struggle to sustain cognitive performance over time during prolonged mental effort. This can be conceptualised as reduced cognitive endurance. If not recognised and addressed, reduced cognitive endurance may limit work participation and delay return to work.

**Methods and analysis:**

This study will compare cognitive endurance, measured as within-day change in cognitive performance, between patients with long-term stress-related disorders treated in primary health care and healthy controls. A secondary aim is to explore how cognitive endurance is associated with self-reported fatigue, daily workload, cognitive function, work ability and work participation. Sixty patients will be recruited from a multicentre trial evaluating work-focused cognitive behavioural therapy in primary health care. An equal number of healthy controls will be recruited through advertisement and approximately matched on age, sex and education. Within-day change in cognitive performance will be assessed through repeating the same battery of cognitive tests in the morning and afternoon of a regular working day. Testing will take place at baseline and 6 months after enrolment, using a cognitive test battery that covers short-term and working memory, attention and processing speed and executive function. The primary outcome will be the morning-to-afternoon change in an overall cognitive composite score, compared between patients and controls. Entropy balancing weights will adjust for residual differences in background characteristics between the two groups, as the control group is non-randomised. Before the assessments, participants will complete questionnaires on trait fatigue, cognitive function, daily workload and work ability. State fatigue will be assessed through self-report questionnaires during the assessment day. For patients, work participation will be measured using sick-leave registry data and self-report. By evaluating a novel method for assessing cognitive endurance in stress-related disorders, the study may provide insights into how variation in cognitive performance over a working day relates to different aspects of fatigue and to functioning in everyday life and at work.

**Ethics and dissemination:**

The study has been approved by the Swedish Ethical Review Authority (Dnr 2025–01713-01; 2025–07089-02). All participants will receive written and oral information about the study and provide written informed consent. Results will be disseminated in peer-reviewed scientific journals, at academic conferences and through communication with key stakeholders.

**Trial registration:**

The study was registered 2 April 2026 at the Open Science Framework (https://doi.org/10.17605/OSF.IO/CYSGX).

**STRENGTHS AND LIMITATIONS OF THIS STUDY**

- Investigating cognitive endurance in a naturalistic setting represents a novel approach to studying cognitive function and its variability that can enhance ecological relevance.
- A well-characterised clinical sample from a primary health care setting increases the generalisability of the findings.
- The design enables exploration of change in cognitive endurance over time (from baseline to 6-month follow-up), while the inclusion of a matched healthy control group provides a reference for interpreting change across repeated measurements.
- The naturalistic setting introduces potential confounding variables. Although efforts will be made to measure, describe and control for such factors, residual confounding may remain.

## Introduction

 Stress-related disorders due to long-term exposure to physical or emotional stressors, classified in the Swedish version of International Classification of Diseases, 10th Revision (ICD-10) as adjustment disorder, exhaustion disorder or reaction to severe stress, are a leading cause of long-term sickness absence in Sweden.^1^ These disorders are associated with a multifaceted symptom profile including fatigue, increased sensitivity to stressors and physical symptoms.^2^ Patients also often report cognitive difficulties, affecting a broad range of areas such as executive function, learning and memory and concentration.^3–8^ Findings from a recent meta-analysis showed that clinical burnout was associated with lower cognitive performance compared with healthy controls in the domains of attention and processing speed, episodic and working memory, executive function and fluency, with small to moderate effect sizes.^9^ In line with this, a previous study found that patients with adjustment disorder or exhaustion disorder (i.e., clinical burnout) performed significantly worse compared with a normative control group across all investigated cognitive domains, including attention and processing speed, executive function and short-term and episodic memory.^10^ The test procedure involved neuropsychological tests that took approximately 20 min to complete and were administered at home at any time during the day, using a digital testing procedure. Across studies, findings have been mixed,^6 7 11–13^ and the relationship between neuropsychological test results and self-reported cognitive difficulties remains unclear.^6 8 10^ This inconsistency may complicate clinical assessment and decisions regarding work ability and return to work.

One possible explanation for this discrepancy is that patients may perform well during a single test session in undisturbed conditions, but fatigue and exhaustion become evident in behavioural performance after extended mental activity. This could reflect reduced cognitive endurance, referring to the capacity to maintain stable cognitive performance over time during prolonged mental effort.^14^ A closely related concept is cognitive fatigability, defined as a quantifiable decline in cognitive performance with sustained time-on-task.^15^ Cognitive endurance can thus be understood as the broader functional capacity to maintain cognitive performance over a workday, whereas cognitive fatigability represents one measurable manifestation of reduced endurance. Patients with long-term stress-related disorders report high levels of mental fatigue, which may be further exacerbated by sustained mental effort.^7 13 16 17^ Mental fatigue and cognitive endurance may therefore be important for understanding fluctuations in cognitive functioning. These constructs may also help explain why standard neuropsychological tests, typically administered under structured and relatively distraction-free conditions, often fail to capture the cognitive difficulties that patients experience in their everyday life. In addition, neuropsychological tests are usually administered at a single time point, which makes it hard to capture fluctuations in performance. A better understanding of the capacity to sustain cognitive performance throughout the day in this patient group may provide valuable information for the treatment and rehabilitation of patients with stress-related disorders.

Cognitive fatigability has been investigated using various methods, including neuropsychological testing^18–20^ and functional brain imaging techniques.^21–23^ However, there is currently no gold standard for its measurement, and how cognitive fatigability manifests in naturalistic, everyday settings remains largely unexplored. Relatedly, a recent proof-of-principle study investigated cognitive endurance over the course of a workday among healthy working adults, demonstrating stable or improved performance following repeated morning and afternoon testing, despite increased levels of self-reported fatigue.^14^ These findings support the feasibility of assessing cognitive endurance through repeated testing over a regular workday, while also emphasising the complex relationship between perceptions of mental fatigue and cognitive performance. From a methodological standpoint, these findings also highlight the need to account for practice effects inherent in repeated cognitive testing^24^ when studying cognitive change.

Clinical experience suggests that patients with stress-related disorders express cognitive difficulties primarily towards the end of the workday. This suggests the need to differentiate between trait fatigue, as a relatively stable symptomatic condition, and state fatigue, as a momentary state-like experience that may fluctuate depending on situational demands.^25^ It remains unclear whether cognitive impairments are a primary symptom of long-term stress-related disorders or are better understood as secondary to fatigue. In the latter case, cognitive capacity would be relatively preserved but become less accessible after exertion. This was investigated in an experimental study that examined the effects of sustained mental activity on perceptions of mental fatigue and cognitive performance. Participants completed a set of cognitive tests before and after a cognitively demanding task with simultaneous auditory exposure.^26^ Results showed that patients diagnosed with exhaustion disorder reported earlier and more persistent increases in mental fatigue than healthy controls, rated the cognitive tasks as more demanding and demonstrated less improvement over time in measures of attention and processing speed, inhibition and working memory. Together, these findings suggest that cognitive difficulties, as experienced by patients with long-term stress-related disorders, may be more pronounced during sustained cognitive demands, for example, at the workplace. Building on this work, we aim to further develop and adapt this procedure for use in a naturalistic setting, in line with the framework for assessing cognitive endurance described by van Vliet *et al*.^14^

In summary, current findings indicate that long-term stress-related disorders may be associated with a reduced ability to sustain cognitive performance over time, together with increased mental fatigue and cognitive difficulties following prolonged mental effort. These findings may have implications for the design and implementation of work-oriented rehabilitation, as reduced cognitive endurance could hinder return to work if not adequately addressed. It has been proposed that cognitive endurance can be assessed through repeated testing over the course of a regular workday,^14^ but empirical research remains limited in both clinical and non-clinical populations. Exploring how daily patterns of cognitive performance relate to fatigue, workload and functioning in everyday life and at work could improve our understanding of cognitive endurance in stress-related disorders and its relevance for occupational functioning.

### Objectives

The objective is to investigate cognitive endurance in individuals with long-term stress-related disorders in a naturalistic setting. Cognitive endurance will be operationalised as within-day change in cognitive performance, measured as the difference between two cognitive assessments conducted on the same working day: one in the morning and one in the afternoon. A healthy control group will be included for comparison. The study aims to:

1. Compare within-day change in cognitive performance between individuals with long-term stress-related disorders and healthy controls at baseline and 6-month follow-up.
2. Investigate the difference in within-day change in cognitive performance from baseline to 6-month follow-up.
3. At each measurement point, examine the associations between within-day change in cognitive performance and the following variables: self-reported trait and state fatigue, perceived workload during the day, number of hours worked, self-reported cognitive function, self-assessed work ability and work participation.

We hypothesise that:

- At baseline, patients with stress-related disorders will show less improvement, or a decline, in within-day change in cognitive performance compared with healthy controls.
- At 6-month follow-up, the difference in within-day change in cognitive performance between patients with stress-related disorders and healthy controls will be smaller than at baseline.

## Methods and analysis

### Study design and setting

This is a study with two measurement points, assessing cognitive endurance in patients with long-term stress-related disorders compared with healthy controls at baseline and 6-month follow-up. For the patient group, baseline assessments will be conducted prior to initiation of work-focused cognitive behavioural therapy (W-CBT), consisting of 5–12 individual sessions delivered in primary health care (PHC). Follow-up assessments will be conducted 6 months after study enrolment. The study is being conducted at PHC centres in the Stockholm Region, Sweden, in collaboration between the Academic Primary Health Care Center and Karolinska Institutet. Data collection commenced in February 2026 and is expected to be completed by the end of 2027, including both baseline and 6-month follow-up assessments.

No more than 2 weeks before baseline testing and 6-month follow-up, participants will be asked to complete questionnaires assessing self-reported trait fatigue, cognitive functioning and work ability, as well as relevant sociodemographic and clinical variables. In addition, state fatigue and relevant contextual factors will be assessed by self-report during the assessment day. Registry data and self-reported sick leave will be used to assess work participation in the patient group. An overview of the procedure is presented in figure 1.

**Figure 1:**
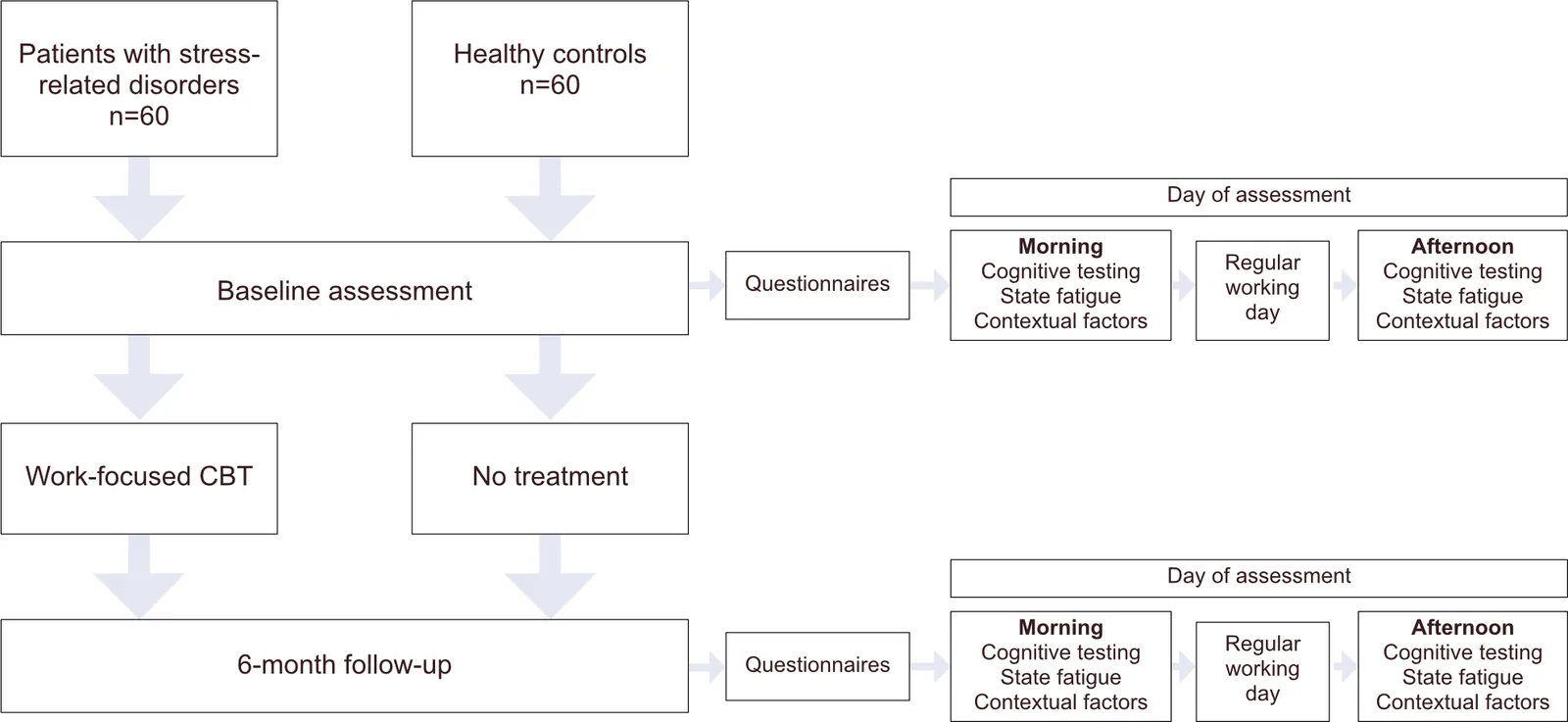
Overview of the study procedure. CBT= cognitive behavioural therapy.

### Participants

#### Patient group

Patients will be recruited from a multicentre intervention study evaluating the effectiveness of W-CBT compared with standard treatment for patients on sick leave or at risk of sick leave due to long-term stress-related disorders in PHC in the Stockholm region; see preregistration at ClinicalTrials.gov: NCT07079709. As part of the intervention study, all participants in the patient group will undergo a structured, in-depth clinical assessment, including a systematic review of the Diagnostic and Statistical Manual of Mental Disorders, Fifth Edition (DSM-5) diagnostic criteria for common mental disorders such as depressive disorders and anxiety disorders. Long-term stress-related disorders will be evaluated based on the diagnostic criteria outlined in the Swedish version of ICD-10 for Adjustment Disorder (F43.2), Exhaustion Disorder (F43.8A), Other Reactions to Severe Stress beyond Exhaustion Disorder (F43.8W), and unspecified reactions to severe stress (F43.9). Patients diagnosed with a primary diagnosis of a depressive disorder, anxiety disorder or any other psychiatric or somatic diagnosis will be excluded.

Patients will be recruited to the present study at the time of inclusion in the intervention study evaluating W-CBT, and all patients included in the present study will receive W-CBT. Healthy controls will be recruited separately and are not part of the intervention study.

Before treatment initiation, a study coordinator will contact interested individuals to provide information about the study, screen for eligibility and answer questions about the study procedures. Those who agree to participate will provide informed consent via a secure digital platform. Following consent, participants will be scheduled for cognitive testing as soon as possible after study inclusion. At baseline, participants may have different working hours because they may be on varying degrees of sick leave. Participants will be recruited consecutively until the target sample size of 60 patient participants is reached. Eligibility criteria are presented in table 1.

**Table 1:** Inclusion and exclusion criteria

Group	Inclusion criteria	Exclusion criteria
Patient group	At risk of sick leave or currently on sick leave. Reports that mental health problems affect functioning at work. Diagnosed with a primary stress-related disorder (F43.2-F43.9), including exhaustion disorder (F43.8A). Scheduled to undergo W-CBT.	Other psychiatric conditions that require prioritisation for immediate or simultaneous treatment: specifically, a current diagnosis of bipolar disorder, schizophrenia, schizoaffective disorder or organic mental disorder; current high risk of suicide; substance use disorder within the past 3 months; previous treatment for stress-related disorders within the past 6 months. Undergoing other psychological treatment.
Control group	Currently employed and working at least 30 hours per week.	Self-reported current or past stress-related or psychiatric disorders. Self-reported current symptoms of exhaustion. Currently on sick leave.
Both groups	Age 18–60 years. Access to an internet-connected computer. Sufficient proficiency in spoken and written Swedish. Provision of informed consent.	Any other medical condition known to affect cognitive function. History of photosensitive epilepsy or migraine.

W-CBT, Work-focused cognitive behavioural therapy.

#### Control group

60 healthy control participants will be recruited through online advertisements distributed via Karolinska Institutet’s social media channels. Interested individuals will register through a secure digital platform where they will provide informed consent and complete a questionnaire covering sociodemographic information, a health declaration and a six-item version of the Shirom-Melamed Burnout Questionnaire (SMBQ).^27^ The SMBQ will be used to screen for symptoms of exhaustion, and individuals scoring above the predetermined cut-off (four points) will be excluded from the study. If there is uncertainty regarding eligibility, a study coordinator will contact the registrant by telephone to conduct a brief screening interview. Individuals who meet the eligibility criteria will be invited to participate. Control participants will be approximately matched to the patient group at the group level based on age, sex and educational level. Eligible participants will be contacted to schedule their first cognitive testing session.

### Procedure

A secure digital platform will be used to register participants and administer questionnaires. Data collection will include a baseline questionnaire covering sociodemographic characteristics (e.g., age, gender, education and living and working conditions) and clinical information (e.g., sick leave status, diagnosis and medication use). In addition, clinical symptoms of burnout, depression and anxiety, as well as fatigue, self-reported cognitive function and work ability, will be assessed at baseline and 6-month follow-up.

Cognitive tests will be administered digitally, and participants will complete them on their own computers at home or at work. Cognitive endurance will be assessed by administering the neuropsychological test battery twice on the same day; once in the morning (08:00–10:00) and once in the afternoon (15:00–17:00). The difference between the two assessments will provide a measure of within-day change in cognitive performance. This procedure will be conducted both at baseline and at the 6-month follow-up. Participants will be instructed to complete the tests on a regular working day, using their own computer in a quiet environment at home or at work, with a stable internet connection.

Each assessment session will begin with a brief video meeting with a test administrator to verify that the testing environment is free from distractions and to allow participants to ask questions about the procedure. During the meeting, the test administrator will also conduct a brief structured interview to assess the participant’s current state, including sleep the previous night, medication use and workload on the day of testing.

Immediately before and after the cognitive tests, participants will complete brief ratings of their current level of mental fatigue, task motivation and perceived performance. These questions will be embedded in the same digital platform as the cognitive test battery.

### Measures

An overview of the study measures, including the constructs assessed, instruments, number of items, response categories and time points for assessments, is presented in table 2.

**Table 2:** Study measures

Construct	Instrument	No. items	Outcome format	Baseline	6MFU	Morning testing	Afternoon testing
Sociodemographic variables							
Sociodemographics	Baseline questionnaire	3		X			
Working conditions	Baseline questionnaire	4		X	X		
Clinical characteristics							
Ongoing sick leave	Baseline questionnaire	1	Yes/no	X	X		
Health and medication	Baseline questionnaire	10		X	X		
Burnout	SMBQ	6	1–7 with all categories labelled	X	X		
Depression	PHQ-9	9	0–4 with all categories labelled	X	X		
Anxiety	GAD-7	7	0–4 with all categories labelled	X	X		
Cognitive functioning							
Attention and processing speed	TMT A	–	Time (s)	X	X		
	TMT D	–	Time (s)	X	X		
	SDPT	–	Correct responses	X	X		
Short-term and working memory	Corsi block tapping test	–	Span length (0–9)	X	X		
Executive function	TMT B	–	Time (s)	X	X		
	Stroop, Index	–	Correct responses/RT (s)	X	X		
	Stroop, Interference	–	RT (ms) Part 2 – Part 1	X	X		
	Verbal fluency (FAS)	–	Correct responses	X	X		
	PASAT	–	Correct responses (0–60)	X	X		
Fatigue							
Trait fatigue	CIS	20	1–7 with all categories labelled	X	X		
State mental fatigue	Items derived from the SMBQ	3	1–6 with all categories labelled			X	X
	Mental effort during testing	1	1–6 with all categories labelled			X	X
	Concentration ability during testing	1	1–5 with all categories labelled			X	X
Self-reported cognitive function							
Cognitive function in relation to lifetime best	Single-item question	1	0–10	X	X		
Self-reported cognitive difficulties	In-house developed questionnaire	27	1–6 with all categories labelled	X	X		
Cognitive function in relation to work	Open-ended questions	8		X	X		
Work ability							
Work functioning	WAI	11		X			
Work functioning	WAS	1	0–10		X		
Work participation				X	X		
Current sick-leave and work percentage	Single item questions	3		X	X		
Sickness absence	Registry data	–					
Contextual and situational factors on day of testing							
Physical environment	Situational interview	4	Yes/no*			X	X
Current state	Situational interview	11	Yes/no*			X	X
Working hours	Situational interview	3	Minutes				X
Workload	NASA-TLX	7	1–5 with all categories labelled†				X
Other variables							
Previous experience of cognitive testing	Situational interview	1	Yes/no			X	
Swedish as a native language	Situational interview	1	Yes/no			X	
Motivation, performance and distractions	Single-item questions	4	Ordinal scale with all categories labelled (range varies)			X	X

More background data are available for the patient group owing to their simultaneous participation in the intervention study evaluating work-focused cognitive behavioural therapy.

* The majority of questions are yes/no, while some have other response options.

† One item: 1–4 with labelled categories.

CIS, Checklist Individual Strength; GAD-7, Generalised Anxiety Disorder Assessment; 6MFU, 6-month follow-up; NASA-TLX, NASA Task Load Index; PASAT, Paced Auditory Serial Addition Test; PHQ-9, Patient Health Questionnaire; RT, reaction time; SDPT, Symbol Digit Processing Test; SMBQ, Shirom-Melamed Burnout Questionnaire; TMT, Trail Making Test; WAI, Work Ability Index; WAS, Work Ability Score.

#### Sociodemographic variables

Information on age, gender, educational level, employment status, occupation, working conditions and current work situation will be collected at baseline.

#### Clinical characteristics

The baseline questionnaire will include questions on previous and ongoing sick leave, as well as general health and other medical diagnoses. Burnout will be assessed using the six-item version of the SMBQ.^27^ The instrument assesses burnout symptoms across two subscales: Emotional exhaustion and physical fatigue, and Cognitive weariness. Depression will be assessed using the Patient Health Questionnaire, a nine-item scale assessing self-reported depressive symptoms over the past 2 weeks.^28^ Anxiety will be assessed using the Generalised Anxiety Disorder Assessment, a seven-item scale assessing generalised anxiety symptoms over the past 2 weeks.^29^

#### Cognitive tests

The cognitive test battery will consist of well-established neuropsychological tests, covering the domains of attention and processing speed, short-term and working memory and executive function. Tests will be administered using Mindmore, a digital cognitive assessment tool in which traditional pencil-and-paper tests have been adapted for digital self-administration.^30^ Mindmore has been validated against analogue testing in healthy adults with satisfactory comparability^30–32^ and acceptable test–retest reliability.^33^ The test battery will comprise nine subtests, with a total administration time of approximately 30 min.

*Attention and processing speed* will be measured using the Trail Making Test (TMT) A and D and the Symbol Digit Processing Test. In TMT, participants are required to connect 25 numbered circles in ascending numbered order as quickly as possible (Part A) and to connect 25 circles in a predetermined order with arrows between the circles (Part D). The outcome measure is completion time in seconds. In the Symbol Digit Processing Test, participants are asked to connect symbols with digits according to a coding key showing nine different symbols, each paired with one of the digits 1–9. The outcome is the number of correct responses in 90 s.

*Short-term and working memory* will be assessed using the Corsi Block-Tapping Test. The test consists of two parts: Corsi Block forward, which targets short-term memory, and Corsi Block backward, which targets working memory. In both conditions, nine squares (blocks) are displayed on the computer screen. A sequence of blocks lights up, and the task is to repeat the sequence, either in the same or reverse order. The first sequence consists of two blocks, and the level of difficulty increases gradually by adding more blocks until the participant makes two consecutive errors at the same level. The outcome is the longest correctly repeated sequence in each condition.

*Executive function* will be assessed using TMT B, the Stroop test, FAS and the Paced Auditory Serial Addition Test. TMT B is a measure of mental flexibility. In this task, the participant is required to connect 25 circles containing digits and letters, alternating between digits and letters in numerical and alphabetical order. The Stroop test is a measure of inhibition and consists of two parts. Twenty colour words (green, yellow, blue or red) are presented one-by-one, either in a congruent (e.g., the word yellow presented in a yellow colour) or incongruent colour (e.g., the word yellow presented in a green colour). The colour words are displayed on four buttons at the bottom of the screen. In the congruent condition, the participant is required to press the button that matches the colour word as quickly as possible. In the incongruent condition, the participant is required to press the button that matches the printed colour of the word as quickly as possible. The outcome is an index score, representing the number of correct answers divided by the average time in seconds in the incongruent condition, and an interference score, representing the average time in milliseconds for the incongruent condition minus the congruent condition. FAS is a measure of verbal fluency. The participant is instructed to say as many words as possible starting with the letters F, A and S. The respondent is given 60 s for each letter, and the total number of correct words across all three trials is used as the outcome. The Paced Auditory Serial Addition Test is a multifactorial task requiring sustained attention, working memory and processing speed. In this task, a series of digits is presented aurally at a rate of one digit every 3 s, and the respondent is required to add each digit to the one previously presented and determine whether the sum is odd or even by pressing a button. The total number of correct answers (maximum 60) is used as the outcome.

#### Fatigue

The Checklist Individual Strength will be used to assess trait fatigue.^34^ The questionnaire consists of 20 items assessing the level of fatigue over the past 2 weeks and comprises four subscales: fatigue severity, concentration problems, reduced motivation and activity. The items are rated on a seven-point ordinal scale, with higher scores indicating more fatigue.

To assess different aspects of state mental fatigue, questions will be administered immediately before and after the cognitive testing. At both time points, participants will rate the current level of mental fatigue and cognitive weariness, using three items derived from the SMBQ (‘I feel mentally tired’, ‘My thinking process is slow’, ‘I have difficulty thinking about complex things’). After completing the cognitive tests, participants will also rate their perceived mental effort and ability to concentrate during the tasks. All items will be rated on a six-point ordinal scale, except for concentration, which will be rated on a five-point scale.

#### Self-reported cognitive function

Self-reported cognitive function will be assessed using a single-item question (‘Assuming that your cognitive function at its best has a value of 10 points. How would you rate your current cognitive function?’), rated on a scale from 0 to 10. Moreover, an in-house developed questionnaire assessing cognitive difficulties in daily life will be administered. The questionnaire consists of 27 items rated on a six-point ordinal scale, covering aspects of attention and processing speed, short-term and working memory, executive function and prospective and retrospective memory. Eight open-ended questions on cognitive function in relation to work will also be included. Four of these concern work accommodations and will be administered only to the patient group.

#### Work ability

Self-assessed work ability will be assessed with the Work Ability Index (WAI)^35 36^ at baseline. This instrument covers seven dimensions: (1) current work ability compared with the lifetime best; (2) work ability relative to job demands; (3) the number of current diseases diagnosed by a physician; (4) estimated work impairment due to those illnesses or injuries; (5) the number of days on sick leave during the past 12 months; (6) personal prognosis of working ability 2 years from now; and (7) general mental resources. The Work Ability Score,^37^ the first item of the WAI, will be used as a follow-up measure. Respondents rate their current work ability compared with lifetime best on a scale from 0 (*cannot work at all*) to 10 (*best work ability*).

#### Work participation

Work participation will be assessed in the patient group through self-report questions on average hours of work per week, work percentage during the past week and current percentage of sick leave, reported as 0%, 25%, 50%, 75% or 100%. Moreover, registry data on sick leave days from day 15 in the sick leave spell will be collected for the patient group from the Swedish Social Insurance Agency database MiDAS and calculated as net sickness absence days. Data will be collected on start and end dates, days on sick pay and sickness and activity allowance, employment status and benefits paid 2 years before inclusion to 5 years after inclusion.

#### Contextual and situational factors on the day of testing

During the interview preceding the cognitive testing, participants will answer questions about contextual and situational factors on the day of testing. These will include questions about the current physical environment (e.g., light, temperature, distractions) and the participant’s current state (e.g., sleep quality last night, alcohol consumption before the testing, current health conditions such as allergies or influenza), and an open-ended question about other life events currently affecting the individual. Moreover, the afternoon interview will include questions about the working situation on the day of testing (e.g., total work hours, hours passed since the participant stopped working), and daytime sleep. To further assess workload on the day of testing, all participants will complete a version of the NASA Task Load Index (NASA-TLX) adapted for the whole day,^38^ before the afternoon testing. The instrument consists of six questions concerning the whole-day workload, across the areas: mental demands, physical demands, temporal demands, effort, performance and frustration. For this project, we adapted the original question ‘How hard did you have to work (with your body or your mind) over your whole day?’, by splitting it into two separate questions: one addressing physical effort and one addressing mental effort.

#### Other variables

We will also collect information on Swedish as a native language, previous experience of cognitive testing, motivation to perform the tests, self-assessed performance on the cognitive tests, and whether any distractions occurred during the cognitive testing.

### Patient and public involvement

There was no patient or public involvement in the planning of this study.

### Data management

During the data collection period, questionnaire data will be collected via two secure research platforms provided by Karolinska Institutet: BASS, a platform from the eHealth Core Facility, and Research Electronic Data Capture (REDCap). Results from the cognitive tests will be collected using the digital testing platform Mindmore. During data collection, the data will be pseudonymised and stored on Mindmore’s data server. Personal identifiers will be stored separately. Register data on sick leave will be obtained from the national register MiDAS using secure linkage procedures. After data collection is complete, all participant data will be pseudonymised and transferred to the secure server at Karolinska Institutet, accessible only to authorised research staff. The final data set will be available to researchers involved in statistical analyses and writing of scientific manuscripts. Data will be electronically stored for the duration necessary to fulfil the research objectives and legal requirements, after which they will be archived or deleted.

### Data analysis plan

Raw scores from the cognitive tests will be transformed to z-scores based on normative values adjusted for age, sex, education and input device (mouse or touchpad). Composite scores will be computed for each cognitive domain (attention and processing speed, short-term and working memory, executive function) by calculating the mean z-score. In addition, an overall cognitive composite score will be computed including all subtests.

Baseline sociodemographic variables, together with symptoms, clinical characteristics and data from the interview preceding the cognitive testing, will be summarised descriptively for each group.

The primary estimand (study aim 1) will be the difference in mean morning-to-afternoon change in the overall cognitive composite between patients with stress-related disorders on, or at risk of, sick leave (SLG) and working controls (CTRL) with the same distribution of pre-existing characteristics as the patients. This will be the average treatment effect in the treated (ATT), with SLG as the group of interest. The ATT is chosen because the study concerns within-day change in cognitive performance in the patient population, and it describes the patients as they are, without reweighting them.

The adjusted difference can be interpreted as the difference attributable to group membership, given the adjustment covariates, under three assumptions.^39^ First, conditional exchangeability: given the adjustment covariates and in the absence of the SLG condition, the two groups would have the same expected change. Second, positivity: each covariate profile that occurs in SLG can also occur in CTRL. The adjusted group difference requires this in one direction, since only CTRL receives varying weights (see below). Third, consistency, which requires a well-defined exposure, which in our study means the group membership.^40^ SLG status can arise in many ways, so the estimand will describe the contrast between the groups as they occur in PHC, not the effect of a specific change to a person’s circumstances. A difference in change between the groups could arise from greater fatigue in SLG or from a smaller practice effect in SLG. The primary estimand will not separate these two explanations.

Weights for CTRL will be estimated by entropy balancing.^41^ SLG participants will receive a weight of 1. Entropy balancing gives exact balance of the covariate means between SLG and weighted CTRL, and combined with a linear outcome model, the estimator is doubly robust for the adjusted group difference.^42^ Covariate balance before and after weighting and the effective sample size of CTRL will be reported. Weight estimation will be conducted separately for the 6-month follow-up sample, since there may be attrition in either group between time points.

Only characteristics that precede the SLG condition will be used for adjustment. These are age, gender, education, employment sector, industry, Swedish as a native language, prior experience of cognitive testing and main job demands. Current symptoms, sleep and medication are consequences or components of the SLG condition, and adjusting for them would therefore remove part of the contrast of interest.

For the outcome regression, the change score will be regressed on group and the adjustment covariates (main effects) in the weighted sample.^43^ The adjusted group difference will then be obtained by g-computation, where each SLG participant’s change will be predicted under both group values, and the difference will be averaged over the SLG participants.^44^ Stantard errors will be computed by M-estimation, which will account for estimation of the weights.^43^

Three descriptive analyses will accompany the primary analysis. The group difference in the morning and afternoon scores will be estimated separately with the same method as the primary estimand (entropy-balancing weights targeting the adjusted group difference, a weighted outcome model including the adjustment covariates and g-computation). These estimates will describe the starting level and the end level of each group and will be reported alongside the difference in change. Floor/ceiling effects for domain and overall composite scores will also be reported. To assess if there is a potential effect of general cognitive ability on change, we will use the three domain component scores as proxy measures of general cognitive ability. The change score of one domain will be regressed on the morning session composite score of the two other domains combined, to investigate if higher ability is associated with a larger change score.

For the comparison between baseline testing and 6-month testing (study aim 2), a stacked analysis will be conducted, where both measurement occasions will be included in the same model. Both the weights regression and the outcome regression will be estimated using measurement occasion in interaction with adjustment covariates. The estimated adjusted group difference for each occasion will be compared with a one-sided hypothesis test that the difference between groups is smaller at the second occasion.

As mentioned under the earlier section ‘Contextual and situational factors on the day of testing’, information about the conditions will be collected through self-report and an interview. A sensitivity analysis will be conducted, omitting participants who report inadequate test conditions.

Sensitivity of the primary estimate to unmeasured confounding will be quantified with the E-value^45^ and with the robustness value of Cinelli and Hazlett.^46^

As secondary analyses, the methods described for the primary estimand will be repeated for each of the three domain composites that make up the overall cognitive composite test score. The three tests will be adjusted for multiplicity with the Holm procedure.

If data from the afternoon session are completely missing, the participant will be excluded from the analyses, because within-day change cannot be estimated. The primary estimand therefore refers to participants who complete both sessions. If data are partially missing at the morning or afternoon session, missing raw scores will be multiply imputed with predictive mean matching. The imputation model will include group, the adjustment covariates, input device and all observed raw scores from both sessions, including the same subtest at the other session. Imputed raw scores will then be transformed to z-scores, and composites and change scores will be computed from the completed data. Twenty datasets will be imputed and analysed with the same pipeline as the primary estimand. Estimates will be pooled with Rubin’s rules.^47^

To examine attrition, participants who complete both morning and afternoon sessions will be compared with those who do not attend the afternoon session. Standardised mean differences will be reported for the adjustment covariates and the morning scores within each group, and a logistic regression of non-completion on group, morning score and their interaction will be fitted. If non-completion is related to the morning score, the primary analysis will be repeated with inverse-probability-of-completion weights.^48^ These analyses can show whether non-completion is related to observed characteristics, but not whether it is related to the unobserved afternoon performance.

Separate regression models that explore linear and potential non-linear associations between one predictor and one outcome (study aim 3) will be performed to investigate the descriptive associations between within-day change in cognitive performance and several variables (separately at baseline and 6 months): self-reported fatigue (state or trait); self-reported cognitive function; self-assessed work ability; work participation; number of working hours on the day of testing; perceived workload during the day, as measured by the NASA-TLX questionnaire. In each regression model, a linear and non-linear relationship between outcome and predictor will be compared, using a natural spline with three degrees of freedom for non-linearity. Whether the non-linear model fits the data better than the linear model will be determined by Akaike’s Information Criterion (AIC), which should be at least two points lower for the non-linear model to be found superior. All predictor variables will be modelled as continuous interval-level variables. To examine whether these associations differ between the two groups, each model will include group and a predictor by group interaction term.

### Sample size calculation

A power analysis was conducted by Monte Carlo simulation (2000 replications per scenario), using the analysis planned for the primary estimand. Based on the results in a previous study,^26^ the target effect was a group difference in morning-to-afternoon change of d=0.4 in normative SD units, and the correlation between morning and afternoon scores was set to intraclass correlation coefficient (ICC)=0.80, with ICC=0.85 as a secondary scenario. To reflect the non-randomised design, the sick leave group was simulated as older, more often female and less educated, with the practice effect allowed to depend on age and education. Each simulated dataset was analysed with entropy-balancing weights targeting the ATT, a weighted outcome model including the adjustment covariates and g-computation, with standard errors that account for estimation of the weights. With 60 participants per group, power was 89% without dropout (94% at ICC=0.85), 83% with 20% dropout in the sick leave group and 10% in the control group (93%), and 79% with 30% and 15% dropout (88%). Simulations were run in R with the packages WeightIt^43^ and marginaleffects.^44^

### Ethics and dissemination

The study has been approved by the Swedish Ethical Review Authority (Dnr 2025–01713-01; 2025–07089-02). All participants will receive oral and written information about the study, including that participation is voluntary, and will provide written informed consent prior to inclusion. Any significant amendments to the protocol will be reported on the study preregistration on the Open Science Framework (https://doi.org/10.17605/OSF.IO/CYSGX). The results will be communicated through scientific articles in peer-reviewed open-access journals, presentations at relevant conferences and directly to key stakeholders, including patients, healthcare professionals in PHC and occupational health services, employers with responsibility for occupational rehabilitation and relevant policymakers. The study will be reported in accordance with the Strengthening the Reporting of Observational Studies in Epidemiology (STROBE) guidelines.

## Discussion

This study will contribute to the understanding of cognitive endurance and return to work in individuals with long-term stress-related disorders by examining within-day changes in cognitive performance in a naturalistic setting and comparing them with those observed in individuals without stress-related disorders. Increased knowledge about cognitive endurance is particularly relevant for this patient group, among whom reduced work ability often leads to sickness absence, which may create barriers that prolong the return-to-work process.^49^ Work capacity assessments for mental disorders are often based largely on patients’ self-reported functioning, which may not always correspond to actual performance capacity. Indeed, self-rated fatigue and perceived cognitive function tend to correlate only weakly with cognitive performance and fatigability,^10 19 50^ indicating that performance-based measures and self-ratings capture different, complementary aspects of functioning.^51^ By investigating a novel procedure for assessing cognitive endurance through repeated cognitive testing, this study may provide valuable insight into cognitive functioning under everyday demands and its relevance for work capacity, thereby contributing to improved assessment procedures.

Cognitive endurance is important for functioning in many occupations in today’s society. Given the relative novelty of the concept, its definition and measurement are still evolving. Cognitive endurance is closely related to cognitive fatigability, which refers to the rate of decline in performance over the course of task execution and is typically measured under controlled conditions.^26^ In contrast, the present study examines cognitive change in a naturalistic setting, operationalising cognitive endurance as within-day change in cognitive performance, between morning and afternoon assessments conducted on the same day. This is consistent with a previous study using a similar testing procedure in a healthy population.^14^ However, changes in cognitive performance over the course of the day may be influenced by several individual and contextual factors, such as fatigue, motivation and daily cognitive demands. Practice effects may also affect performance on repeated testing. Although the inclusion of a healthy control group undergoing the same measurement procedure provides an important reference point for interpretation, additional factors such as age and clinical status may still influence the magnitude of practice effects.^24^ Taken together, the difference in cognitive performance between morning and afternoon testing should not be interpreted as a direct measure of cognitive endurance, but rather as an exploratory indicator of the construct. Given the novelty of the field, this approach may nevertheless provide new insights into how cognitive endurance can be conceptualised and measured, as well as into factors associated with daily variability in cognitive performance.

The present study uses a longitudinal controlled study design to investigate how cognitive endurance changes over time in patients with long-term stress-related disorders. Many occupations involve tasks that place substantial demands on cognitive capacity, yet few studies have investigated the longitudinal course of cognitive functioning in long-term stress-related disorders,^52^ or its relationship with functional outcomes. Although self-reported cognitive concerns may predict future sick leave,^53^ and lower fatigue and better cognitive performance have been associated with return to work in other patient groups,^54^ the relationship among these symptoms, cognitive endurance and the return-to-work process in patients with stress-related disorders remains largely unexplored. This study does not aim to evaluate treatment effects over time, but rather to assess changes in sustained cognitive performance across both short- and long-term intervals in the context of daily mental and physical demands. In doing so, it may provide insights into how variation in cognitive functioning relates to work capacity and performance. These findings may have clinical implications for the design of rehabilitation interventions and for how the workplace can be used to support cognitive functioning.

### Strengths and limitations

This study should be interpreted in the light of several methodological strengths and limitations. A major strength is its naturalistic setting, which enhances ecological relevance, as laboratory-based exposure may not adequately capture the demands patients experience in daily life. Participants will also complete the cognitive tests remotely, reducing the burden of clinic visits and enabling assessment of cognitive performance in an everyday context. This is particularly relevant because assessment of work ability in relation to mental health problems often relies on patients’ reports of functioning in daily life, making the everyday context central to interpretation. Furthermore, workload will be assessed quantitatively regardless of whether the participant is currently working or on sick leave, with the aim of capturing their total daily load. In addition, the sample will include patients from PHC, who will undergo a systematic assessment procedure to support diagnostic accuracy, as fatigue may occur in a range of mental and somatic disorders.^55^ Both trait and state fatigue will be assessed using separate measures, allowing changes in state fatigue during the day to be captured alongside broader fatigue levels at baseline and at 6-month follow-up. In addition, fatigue-related functioning will be assessed using both self-report questionnaires and cognitive tests, in line with recommendations to capture the multidimensional nature of the construct.^15 56^ To reduce participant burden, the cognitive test battery was limited to domains previously demonstrated to be affected by long-term stress^9^ and mental fatigue.^26^ However, this targeted selection limits the study’s ability to provide a comprehensive profile of cognitive functioning.

Despite the strengths of the naturalistic setting, the remote testing procedure also has limitations. Reduced control over the testing situation may introduce confounding factors and affect performance validity. For example, inadequate motivation, changes in the testing environment between the morning and afternoon assessments, or unexpected interruptions may influence test performance. However, in this research context, test results have no direct clinical, occupational or financial consequences. Some studies suggest that invalid performance is less common when clear external incentives are absent,^57^ although this does not preclude invalid or suboptimal performance.^58^ However, the structured interview conducted during the video meeting before testing is intended to identify contextual and individual factors to ensure adequate testing conditions. Interview data will be described separately for each group, and sensitivity analyses will be conducted if inadequate testing conditions are identified. Standalone performance validity tests were not available within the digital test battery, which may limit the ability to evaluate performance validity beyond indirect indicators such as task completion, reported distractions and structured pretest checks.

Further, given the naturalistic setting, daytime workload will vary both within and between groups. Differences in sick leave duration, working conditions and the possibility of work accommodations may therefore influence the outcomes of interest. Such variation is inherent to the naturalistic setting and to the populations being compared. To support interpretation, relevant contextual factors will be documented and summarised descriptively, and potential confounders will be accounted for in the statistical analyses when appropriate.

The diagnosis of adjustment disorder has several limitations. Despite more than 50 years in diagnostic classifications and frequent use in clinical practice, its criteria remain vague and poorly operationalised. It also retains a subthreshold status, meaning it can only be assigned when criteria for another mental disorder are not met. Adjustment disorder has further been criticised for serving as a residual category and for pathologising normal stress reactions.^59^ We will therefore conduct a careful differential diagnostic assessment to rule out other primary diagnoses, and, to ensure clinical relevance, include only individuals with clear functional impairment, such as ongoing sick leave or work-related functional impairment. However, the exploratory design of the study limits generalisability to other healthcare systems, clinical populations or settings.
