# Supplementary material for: Cognitive function and return to work: protocol for a longitudinal study investigating cognitive endurance in patients with stress-related disorders in primary health care in Sweden
Source: BMJ Open. 2026 Nov 6;16(11):e125346. doi: 10.1136/bmjopen-2026-125346 (PMC13756220; doi:10.1136/bmjopen-2026-125346)
Supplement: Supplementary file 1 [file bmjopen-2026-125346.reviewer_comments.pdf]

PEER REVIEW HISTORY

BMJ Open publishes all reviews undertaken for accepted manuscripts. Reviewers are asked to complete a checklist review form and are provided with free text boxes to elaborate on their assessment. These free text comments are reproduced below.

ARTICLE DETAILS

Title (Provisional)

Cognitive Function and Return to Work: Protocol for a Longitudinal Study Investigating Cognitive Endurance in Patients with Stress-Related Disorders in Primary Health Care

Authors

Malmberg, Hanna; Zakrevska, Anastasiya; Johansson, Magnus; Nordberg, Anna; Björk Brämberg, Elisabeth Björk; van Vliet, Fabienne I.M.; Donker-Cools, Birgit; Schaafsma, Frederieke; Finnes, Anna

---

VERSION 1 - REVIEW

**Reviewer**	**1**
**Name**	**Gerstenecker, Adam**
**Affiliation**	**UAB, Neurology**
**Date**	**23-Aug-2026**
**COI**	**None**

The study protocol “Cognitive Function and Return to Work: Protocol for a Longitudinal Study Investigating Cognitive Endurance in Stress-Related Disorders” describes a longitudinal investigation of “cognitive endurance” in individuals with long-term stress-related disorders using repeated cognitive testing across a typical workday. The topic is potentially interesting, particularly given the discrepancy that can occur between subjective cognitive complaints and performance during conventional cognitive testing. The more real-world approach and longitudinal follow-up are also potentially useful. However, I have several concerns regarding the conceptualization and operationalization of cognitive endurance, characteristics of the clinical and control groups, interpretation of repeated cognitive testing, and the proposed statistical analyses. These and other concerns are discussed below:

• The authors define cognitive endurance as the difference between morning and afternoon cognitive performance, adjusted for workload during the day. However, change across two administrations of the same cognitive measures may be influenced by a number of other factors including practice effects, sleep/fatigue, and activities occurring between assessments. The manuscript does not adequately establish that the resulting difference score specifically represents “cognitive endurance.” This is particularly important because the primary hypothesis considers both declining performance and smaller practice effects to represent reduced cognitive endurance. A smaller improvement with repeated testing is not necessarily equivalent to deterioration in cognitive performance. The conceptual and psychometric basis for treating these outcomes as similar is in need of considerably more support.

- Relatedly, the authors suggest that inclusion of a control group will help mitigate practice effects. Practice effects can vary according to baseline cognitive ability, clinical status, prior exposure, and other individual factors. The authors should more carefully describe how practice effects will be modeled and explain the assumptions required to interpret differential improvement between groups as evidence of reduced cognitive endurance.

- Another major concern involves differences in what occurs between the morning and afternoon assessments. The study is intended to evaluate cognitive endurance under real-world conditions, but patients may differ considerably in real-world factors such as work status, hours worked, and work-related activities. Thus, participants are not necessarily exposed to comparable cognitive or occupational demands between assessments. The authors propose measuring workload and statistically adjusting for these differences, but statistical adjustment does not make the underlying exposures equivalent. In addition, differences in daytime/work activity may be particularly problematic when the primary outcome is change in cognitive performance across that same period.

- The clinical sample is also heterogeneous. “Long-term stress-related disorders” includes Adjustment Disorder, Exhaustion Disorder, other reactions to severe stress, and unspecified reactions to severe stress. The manuscript does not provide sufficient justification for treating these diagnoses as a single clinical population for purposes of cognitive analysis. This concern is particularly relevant because fatigue and cognitive complaints may differ across these conditions and may also be influenced by co-occurring depression and anxiety. At minimum, the authors should more clearly justify this grouping and describe how diagnostic and psychiatric heterogeneity will be addressed analytically.

- The recruitment strategy also raises concerns regarding interpretation and generalizability. Patients are recruited in the context of sick leave for long-term stress-related disorders, whereas healthy controls are required to be actively employed. Consequently, the groups differ in daily activity. This is particularly important because work ability and return to work are among the outcomes of interest. The manuscript should discuss the degree to which this selection strategy may influence observed associations and should more clearly address the generalizability of findings beyond the Swedish healthcare system.

- I also have concerns regarding statements about change “following treatment.” Patients undergo W-CBT between baseline and six-month follow-up, but this cognitive study does not appear to include a comparable clinical group that does not receive this intervention. Therefore, improvement from baseline to follow-up cannot be attributed specifically to treatment and any changes could reflect a number of factors.

- Performance validity represents another limitation. Cognitive testing will occur remotely in a real-world setting without designated performance validity measures. The authors acknowledge this limitation, but it is particularly important when the primary dependent variable is within-person change across repeated assessments. This issue deserves greater emphasis when discussing interpretation of the findings.

- Finally, the Discussion is underdeveloped for a study with this degree of conceptual and methodological complexity. It primarily reiterates the potential importance and clinical implications of the study. The Discussion should more directly address the construct validity of

cognitive endurance, interpretation of practice effects, heterogeneity of the clinical sample, differences in daytime exposure between groups, limitations on causal interpretation of longitudinal change, and generalizability of this healthcare and occupational framework to other settings. Claims that the real-world setting "ensures high ecological validity" should also be tempered. A "naturalistic" setting may increase ecological relevance, but it reduces experimental control and does not establish ecological validity.

• More generally, the manuscript requires enhanced editing. There are frequent awkward or unclear sentences, grammatical problems, and inconsistent use of related terms such as cognitive endurance, cognitive fatigability, sustained performance, work endurance, cognitive capacity, and work capacity. Greater terminological precision would also help clarify exactly what construct the authors intend to measure.

VERSION 1 - AUTHOR RESPONSE

<u>**Reviewer: 1**</u>

Dr. Adam Gerstenecker, UAB
Comments to the Author:

The study protocol "CogniCve FuncCon and Return to Work: Protocol for a Longitudinal Study InvesCgaCng CogniCve Endurance in Stress-Related Disorders" describes a longitudinal invesCgaCon of "cogniCve endurance" in individuals with long-term stress-related disorders using repeated cogniCve tesCng across a typical workday. The topic is potenCally interesCng, parCcularly given the discrepancy that can occur between subjecCve cogniCve complaints and performance during convenConal cogniCve tesCng. The more real-world approach and longitudinal follow-up are also potenCally useful. However, I have several concerns regarding the conceptualizaCon and operaConalizaCon of cogniCve endurance, characterisCcs of the clinical and control groups, interpretaCon of repeated cogniCve tesCng, and the proposed staCsCcal analyses. These and other concerns are discussed below:

#1 The authors define cogniCve endurance as the difference between morning and aZernoon cogniCve performance, adjusted for workload during the day. However, change across two administraCons of the same cogniCve measures may be influenced by a number of other factors including pracCce effects, sleep/faCgue, and acCviCes occurring between assessments. The manuscript does not adequately establish that the resulCng difference score specifically represents "cogniCve endurance." This is parCcularly important because the primary hypothesis considers both declining performance and smaller pracCce effects to represent reduced cogniCve endurance. A smaller improvement with repeated tesCng is not necessarily equivalent to deterioraCon in cogniCve performance. The conceptual and psychometric basis for treaCng these outcomes as similar is in need of considerably more support.

Authors' response: The morning–abernoon approach is not completely new but builds on the previous proof-of-principle study in healthy workers (DOI: 10.1007/s10926-025-10346-8), and therefore the current defini7on was ul7mately adopted. Our use of the term "cogni7ve endurance" in this manuscript is only meant to describe the (poten7al) within-day change in cogni7ve performance in our quasi-experimental design. We do not claim it to be a defini7on of the construct in a broader sense. Throughout the manuscript, we have now changed the term "cogni7ve endurance" to "within-day change in cogni7ve performance" when referring to the actual measurement.

We have rewrieen our defini7on of the study's primary es7mand, which should clarify what we aim to assess and how we plan to do so, including the assump7ons we make (page 21, rows 421-436). While we agree that the explana7on for the poten7al finding that the sick-leave group (SLG) shows a significantly lower within-day change score is not necessarily to be interpreted as "deteriora7on in cogni7ve performance", it is one of several possible interpreta7ons of such a result.

Our hypotheses were informed by previous studies using prolonged tes7ng procedures to examine cogni7ve fa7gability in controlled seEngs, in which pa7ents with mental fa7gue have been found to show less improvement in performance across repeated tes7ng compared to healthy controls (DOI: 10.1097/01.HTR.0000308719.70288.22; DOI: 10.3389/fnhum.2019.00145; DOI: 10.1002/brb3.1056). A similar paeern has been observed for pa7ents with stress-related disorders, as outlined in the introduc7on (DOI: 10.1016/j.biopsycho.2023.108661). However, we agree that such a paeern should not be interpreted as a directly represen7ng reduced cogni7ve endurance and have therefore clarified our hypothesis to focus on within-day change in cogni7ve performance; see page 9, rows 176-179. In addi7on, we have elaborated the Discussion on the conceptualiza7on and measurement of cogni7ve endurance; see page 26, rows 547-563.

#2 Relatedly, the authors suggest that inclusion of a control group will help miCgate pracCce effects. PracCce effects can vary according to baseline cogniCve ability, clinical status, prior exposure, and other individual factors. The authors should more carefully describe how pracCce effects will be modeled and explain the assumpCons required to interpret differenCal improvement between groups as evidence of reduced cogniCve endurance.

Authors' response: As cogni7ve endurance in pa7ents with long-term stress-related disorders has not yet been explored, we believe that a reasonable first step is to examine whether paeerns of cogni7ve performance across a regular working day differ between pa7ents and healthy controls. The control group is included as a reference group to provide a range of reasonably expected prac7ce effects in a group of healthy individuals.

On page 5, rows 76-78, we now write: "The design enables explora7on of change in cogni7ve endurance over 7me (from baseline to six-month follow-up), while the inclusion of a matched healthy control group provides a reference for interpre7ng change across repeated measurements"

As noted in the previous response, we have revised our descrip7on of the primary es7mand and the assump7ons we make, connec7ng the es7mand to the es7mator chosen. The power analysis was also updated to reflect the planned analysis method; see page 25, rows 506-518.

There may indeed be unmeasured confounders, and we plan to run a sensi7vity/robustness analysis using the E-value method (also added to the Methods sec7on, page 23, rows 472-473). We expect that the morning measurement (assumed to be what "baseline cogni7ve ability" refers to) will be different between groups. We will evaluate and report this descrip7vely, together with the poten7al associa7on between morning session performance and change, to help aid interpreta7on of results (more details are in the rewrieen Methods sec7on). We will also inves7gate if there are floor/ceiling effects at the morning session that could limit poten7al change.

We have added a sec7on to the Discussion addressing the use of a control group (page 27, rows 555-563).

#3 Another major concern involves differences in what occurs between the morning and aZernoon

assessments. The study is intended to evaluate cogniCve endurance under real-world condiCons, but paCents may differ considerably in real-world factors such as work status, hours worked, and work-related acCviCes. Thus, parCcipants are not necessarily exposed to comparable cogniCve or occupaConal demands between assessments. The authors propose measuring workload and staCsCcally adjusCng for these differences, but staCsCcal adjustment does not make the underlying exposures equivalent. In addiCon, differences in dayCme/work acCvity may be parCcularly problemaCc when the primary outcome is change in cogniCve performance across that same period.

Authors' response: We agree with the reviewer that problems arise from the differences in exposure between groups. However, differences in what people do during the day are largely part of the naturalis7c seEng and of the popula7ons we are comparing. Workload could be thought of as a level of demand or context under which cogni7ve performance is tested, rather than a confounder that should be adjusted for. The ques7onnaires and day-specific assessments should help us capture that context, but of course they do not make the exposure the same. Workload may influence how fa7gued someone feels, but feeling more fa7gued does not necessarily mean that cogni7ve performance gets worse. We do not necessarily expect a simple rela7onship in which a higher workload leads to poorer cogni7ve performance.

We have added a specific aim of examining the associa7on between workload and within-day change in cogni7ve performance, and the analysis plan has been revised accordingly (page 24, rows 493-505). This will allow us to examine more directly the extent to which workload (both working hours and perceived workload during the day) contributes to varia7on in cogni7ve performance across the day.

Our third aim (page 9, rows 171–174) is now formulated as follows: 3) At each measurement point, examine the associations between within-day change in cognitive performance and the following variables: self-reported trait and state fatigue, perceived workload during the day, number of hours worked, self-reported cognitive function, self-assessed work ability, and work participation.

#4 The clinical sample is also heterogeneous. "Long-term stress-related disorders" includes Adjustment Disorder, ExhausCon Disorder, other reacCons to severe stress, and unspecified reacCons to severe stress. The manuscript does not provide sufficient jusCficaCon for treaCng these diagnoses as a single clinical populaCon for purposes of cogniCve analysis. This concern is parCcularly relevant because faCgue and cogniCve complaints may differ across these condiCons and may also be influenced by co-occurring depression and anxiety. At minimum, the authors should more clearly jusCfy this grouping and describe how diagnosCc and psychiatric heterogeneity will be addressed analyCcally.

Authors' response: We agree that diagnos7c criteria in stress-related disorders partly overlap with other psychiatric diagnoses. In the interven7on study, all par7cipants undergo a thorough diagnos7c procedure, including differen7al diagnosis. Pa7ents diagnosed with a primary diagnosis of depression, an anxiety disorder or another psychiatric or soma7c diagnosis are excluded. Only pa7ents with a primary diagnosis of a stress-related disorder according to the Swedish version of ICD-10 (F43.2-F43.9) and pronounced func7onal disability related to the diagnosis will be included. We argue that these measures sufficiently exclude pa7ents with other primary mental disorders and that the stress-related disorders have common features such as fa7gue, cogni7ve complaints and intolerance to stress.

A study with a similar primary care popula7on found no significant differences in cogni7ve performance between par7cipants diagnosed with adjustment disorder and exhaus7on disorder (DOI: 10.1186/s12888-023-05048-5). Both groups performed significantly worse than healthy controls.

We have made clarifica7ons in the Introduc7on, page 6, rows 85-89 and in the Methods sec7on, page 11, rows 213-214. In addi7on, we have included a sec7on in the Discussion (page 29, rows 619-627) concerning the limita7ons of the adjustment disorder diagnosis.

#5 The recruitment strategy also raises concerns regarding interpretaCon and generalizability. PaCents are recruited in the context of sick leave for long-term stress-related disorders, whereas healthy controls are required to be acCvely employed. Consequently, the groups differ in daily acCvity. This is parCcularly important because work ability and return to work are among the outcomes of interest. The manuscript should discuss the degree to which this selecCon strategy may influence observed associaCons and should more clearly address the generalizability of findings beyond the Swedish healthcare system.

Authors' response: The difference between groups in their daily ac7vity is difficult to avoid, because an ac7vely working healthy control group provides the most relevant reference for cogni7ve func7oning across a workday. The contrast in work status is therefore partly built into the study ques7on. At the same 7me, it does mean that between-group differences may partly reflect differences in daily work par7cipa7on and ac7vity, so this will be acknowledged in the interpreta7on of the results.

We would also like to clarify that the outcome concerning return to work is only of interest in the pa7ent group, since the inclusion criteria for the healthy controls are that they are currently working. We have changed the wording from "return to work" to "work par7cipa7on" to beeer fit with the pa7ent group, which includes both sick-listed and working individuals at risk of sick leave. We agree that par7cipants work par7cipa7on can be partly affected by the healthcare and insurance system in which it is studied, and this has been added to the Discussion (page 29, rows 626-627).

#6 I also have concerns regarding statements about change "following treatment." PaCents undergo W-CBT between baseline and six-month follow-up, but this cogniCve study does not appear to include a comparable clinical group that does not receive this intervenCon. Therefore, improvement from baseline to follow-up cannot be adributed specifically to treatment and any changes could reflect a number of factors.

Authors' response: It is correct that this study cannot provide informa7on on cogni7ve improvement as an effect of treatment due to the lack of a clinical group not receiving W-CBT. This study is a longitudinal follow-up, which allows us to examine whether cogni7ve performance changes during the study period. To mi7gate the risk of interpre7ng the aim as examining causality, we have revised the wording so that it does not imply that W-CBT caused the observed changes. The statement "following treatment" has been replaced with the measurement point of "six-month follow-up".

For example, on page 9, rows 169-170, we write: "Inves7gate the difference in within-day change in cogni7ve performance from baseline to six-month follow-up"

#7 Performance validity represents another limitaCon. CogniCve tesCng will occur remotely in a real-world se<ng without designated performance validity measures. The authors acknowledge this limitaCon, but it is parCcularly important when the primary dependent variable is within-person change across repeated assessments. This issue deserves greater emphasis when discussing interpretaCon of the findings.

Authors' response: First, we would like to clarify that the primary dependent variable in our analyses is between-group differences in change. Second, a brief interview including ques7ons about the test environment and current state will be conducted at each tes7ng session to establish the seEng in which the individual is doing the tests. The informa7on obtained will be presented descrip7vely and sensi7vity analyses will be conducted, see updated Methods sec7on for details (page 23, rows 469-471).

S7ll, performance validity should receive more aeen7on. We believe that since the results do not have direct clinical, occupa7onal or financial consequences, it may be less likely to underperform than in high-stakes seEngs. This has now been highlighted more in the Discussion sec7on (page 28, rows 600-612), and two references have been added to support the argumenta7on (DOI: 10.1080/13854046.2021.1895322; DOI: 10.1007/s11065-023-09582-7).

#8 Finally, the Discussion is underdeveloped for a study with this degree of conceptual and methodological complexity. It primarily reiterates the potenCal importance and clinical implicaCons of the study. The Discussion should more directly address the construct validity of cogniCve endurance, interpretaCon of pracCce effects, heterogeneity of the clinical sample, differences in dayCme exposure between groups, limitaCons on causal interpretaCon of longitudinal change, and generalizability of this healthcare and occupaConal framework to other se<ngs. Claims that the real-world se<ng "ensures high ecological validity" should also be tempered. A "naturalisCc" se<ng may increase ecological relevance, but it reduces experimental control and does not establish ecological validity.

Authors' response: We have elaborated on the raised issues in the Discussion sec7on by adding a sec7on on the construct validity of cogni7ve endurance (page 26, rows 547-563), the interpreta7on of the longitudinal study design (page 27, rows 572-575), and as previously men7oned, prac7ce effects (page 27, rows 555-563), and the clinical sample and the generalisability of the findings (page 29, rows 619-627). For issues already men7oned in the Discussion, we have elaborated and clarified them. Finally, we agree that the term "ecological relevance" is more accurate than "ecological validity" and have revised the wording in the manuscript accordingly.

#9 More generally, the manuscript requires enhanced ediCng. There are frequent awkward or unclear sentences, grammaCcal problems, and inconsistent use of related terms such as cogniCve endurance, cogniCve faCgability, sustained performance, work endurance, cogniCve capacity, and work capacity. Greater terminological precision would also help clarify exactly what construct the authors intend to measure.

Authors' response: It should be noted that there are differences in terminology within the research field, which makes it challenging to be both consistent and accurate. We have gone through the manuscript and revised it to achieve beeer consistency in the terminology. In addi7on, the manuscript has been revised throughout using Claude (Anthropic) for proofreading to improve grammar, phrasing, and sentence structure.

Reviewer: 1
If you have selected ‘Yes’ above, please provide details of any compe7ng interests.: Not applicable

VERSION 2 - REVIEW

**Reviewer**	**1**
**Name**	**Gerstenecker, Adam**
**Affiliation**	**UAB, Neurology**
**Date**	**17-Oct-2026**
**COI**	

The authors have provided thoughtful and substantive responses to my previous comments, and the revised manuscript is considerably improved. In particular, I appreciate the clearer distinction between within-day changes in cognitive performance and the broader construct of cognitive endurance, as well as the more cautious interpretation of practice effects and treatment-related changes. The expanded discussion of methodological limitations is also helpful.

I have one remaining concern regarding the description and interpretation of the statistical approach. The authors now provide additional detail about entropy balancing and the average treatment effect on ATT. However, given the observational design and the differences between the clinical and healthy control groups, I recommend the avoidance of terminology that could imply a causal effect of patient-groups. The analyses are more appropriately interpreted as adjusted between-group comparisons, and I recommend ensuring that this distinction is maintained throughout the manuscript.

Otherwise, I believe the authors have adequately addressed my previous concerns, and I have no additional substantive recommendations.

VERSION 2 - AUTHOR RESPONSE

Reviewer: 1
Dr. Adam Gerstenecker, UAB
Comments to the Author:
The authors have provided though@ul and substanCve responses to my previous comments, and the revised manuscript is considerably improved. In parCcular, I appreciate the clearer disCncCon between within-day changes in cogniCve performance and the broader construct of cogniCve endurance, as well as the more cauCous interpretaCon of pracCce effects and treatment-related changes. The expanded discussion of methodological limitaCons is also

helpful.
I have one remaining concern regarding the descripCon and interpretaCon of the staCsCcal approach. The authors now provide addiConal detail about entropy balancing and the average treatment effect on ATT. However, given the observaConal design and the differences between the clinical and healthy control groups, I recommend the avoidance of terminology that could imply a causal effect of paCent-groups. The analyses are more appropriately interpreted as adjusted between-group comparisons, and I recommend ensuring that this disCncCon is maintained throughout the manuscript.
Otherwise, I believe the authors have adequately addressed my previous concerns, and I have no addiConal substanCve recommendaCons.

Authors' response:

We thank the reviewer for this comment. We agree that the results should be described in a way that does not imply that belonging to the patient group, as such, causes the observed differences. Group membership is not an intervention; the groups were not assigned, and although we have aimed to measure the most important confounders, no observational design can guarantee that the adjustment set is complete. We have revised the manuscript throughout so that the results are described as covariate-adjusted between-group differences.

We have retained the formal descripBon of the estimand and the assumptions it relies on, because we believe this makes the limitations of the comparison explicit rather than implicit. The term average treatment effect in the treated (ATT) is now used only twice, in the Methods describing the entropy weighBng procedure and the power analysis, to name the target populaBon of the weighBng (the paBent group). We explain there that it denotes the adjusted difference between the paBent group and controls reweighted to the paBents' distribuBon of age, gender and educaBon. The assumpBons secBon states that the adjusted difference could only be interpreted causally if there were no unmeasured confounding, if every covariate profile in the paBent group also occurred among controls, and if the exposure (group membership) were well defined. The sensiBvity analyses for unmeasured confounding (E-value and robustness value) are retained and are described as quanBfying how strong unmeasured confounding would need to be to account for the adjusted difference.
